# Switching on the evolutionary potential of pancreatic cancer: the tumor suppressor functions of *PBRM1*

**DOI:** 10.1172/JCI193205

**Published:** 2025-06-02

**Authors:** Luigi Perelli, Giannicola Genovese

**Affiliations:** 1 Department of Genitourinary Medical Oncology,; 2 TRACTION platform, and; 3 Department of Genomic Medicine, UT MD Anderson Cancer Center, Houston, Texas, USA.

## Abstract

Cell plasticity is a hallmark of cancer, enabling tumor cells to acquire multiple phenotypes responsible for tumor progression, metastasis, and therapy resistance. In this issue of the *JCI*, Kawai and colleagues leveraged genetically engineered mouse models (GEMM) of pancreatic ductal adenocarcinoma (PDAC) to demonstrate that loss of *Pbrm1*, a member of the SWI/SNF complex, drives dedifferentiation and aggressive tumor features. *Pbrm1* loss activated a program of epithelial-to-mesenchymal transition (EMT) and allowed the emergence of poorly differentiated histologies that are commonly associated with high recurrence rate and dismal prognosis. These findings reveal the role of the SWI/SNF complex during PDAC evolution in maintaining cell identity and restraining the progression of this lethal disease.

## An alternative preclinical model of PDAC

**Figure 1:**
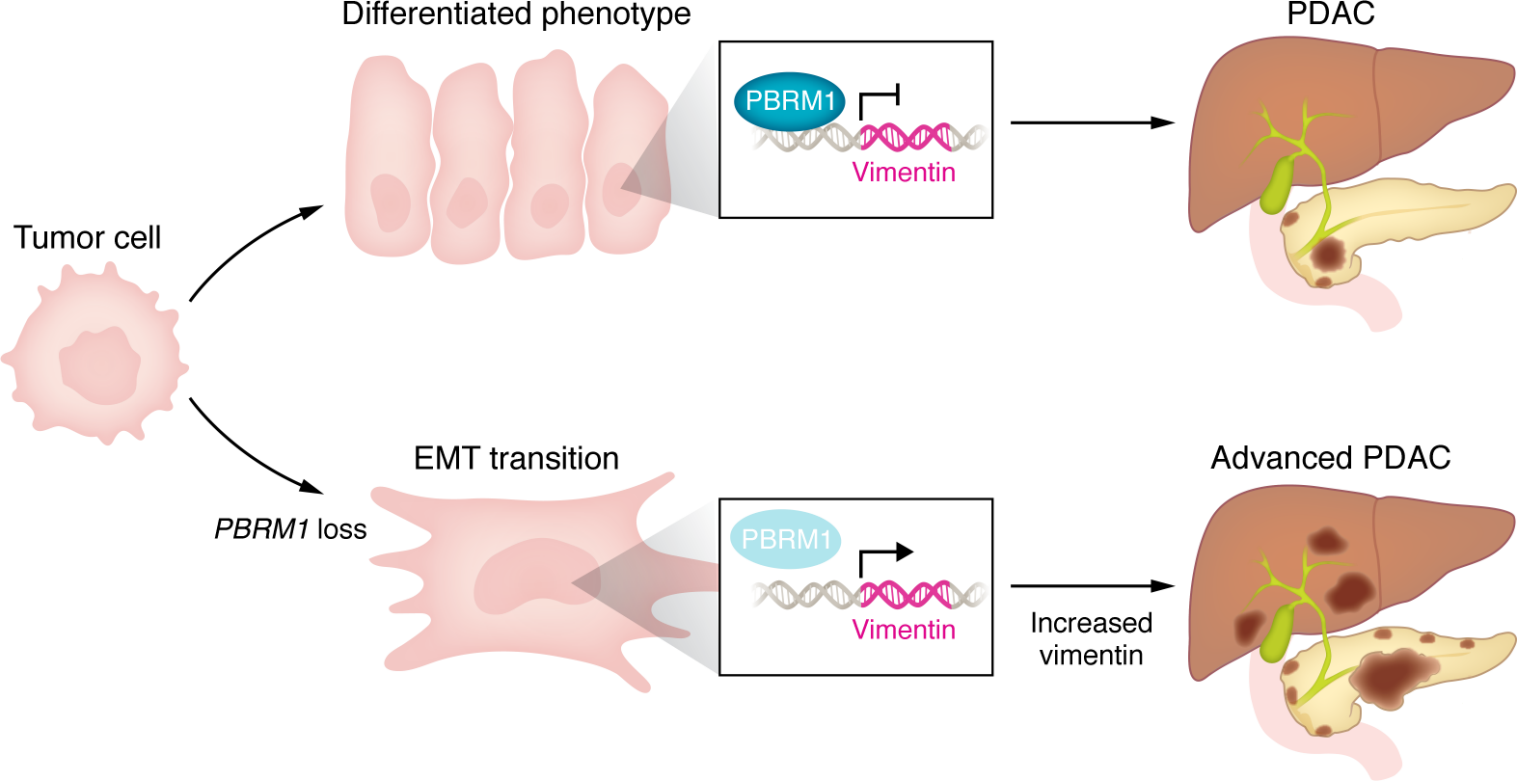
PBRM1 is a gatekeeper of PDAC evolutionary potential. PBRM1 has a role in maintaining a ductal identity in PDAC. Conversely, PBRM1 loss leads to disease progression and the positive selection of undifferentiated cancer cells. PBRM1 binds directly to the promoter of the EMT mediator vimentin, which is upregulated with PBRM1 loss.

Pancreatic cancer is the third leading cause of cancer-related death in the US and has the highest mortality rate (1). The high lethality of PDAC is mainly caused by the prominent capacity of malignant cells to invade and metastasize early on, during the natural history of the disease (2). Furthermore, very few therapeutic options are available for this tumor type, most of which are based on a combination of cytotoxic chemotherapy and radiation (2). This clinical scenario is mainly sustained by the emergence of tumor cells with a high degree of cellular plasticity, promoting adaptation and intratumor heterogeneity during PDAC progression (2–5). Solid experimental evidence has highlighted genetic and nongenetic disregulation of the SWI/SNF chromatin remodeling complex as a key biomolecular driver of PDAC aggressive behavior and metastasis by virtue of its ability to regulate lineage specification and maintenance of terminal differentiation programs (6). In this issue of the *JCI*, Kawai et al. (7) developed robust in vivo models to study the role of *Pbrm1*, a poorly characterized member of the SWI/SNF family, and part of the polybromo-associated BRG1/BRM-associated factor (PBAF) subunit, in pancreatic cancer. The authors generated a *Pbmr1*-floxed allele to investigate its inactivation during PDAC tumorigenesis and progression in a *Kras^G12D^* and *Kras^G12D^/Trp53^fl^* mutant background. Indeed, they demonstrated that loss of *Pbrm1* synergized with oncogenic *Kras* and *Trp53* deletion during tumorigenesis and tumor progression, providing a growth advantage (Figure 1). Moreover, inactivation of *Pbrm1* in advanced tumors resulted in the emergence of high-grade histologies characterized by dedifferentiation toward adenosquamous and/or sarcomatoid phenotypes. Such morphological variants are associated in patients’ datasets with poor prognosis and aggressive disease (8). These results advance our knowledge of the role of the SWI/SNF complex in PDAC tumor progression and add a layer of complexity to the effects of dysfunctional chromatin remodeling to solid tumor progression (6, 9). From a therapeutic standpoint, Kawai and colleagues demonstrated that this aggressive PDAC variant with *Pbrm1* loss is sensitive to depletion of the intermediate filament Vimentin, a critical marker of EMT (5, 7). Intriguingly, these findings have been also recapitulated by our group in preclinical models of *SMARCB1-* and *ARID1A*-altered PDAC, further supporting the notion that EMT is required for the emergence and maintenance of aggressive cancer cell subpopulations and further suggesting dysregulation of the SWI/SNF complex function as a potent driver of mesenchymal plasticity (5, 6, 9).

## SWI/SNF regulates intratumor heterogeneity

To understand the effects of *Pbrm1* loss on cell plasticity and dedifferentiation, Kawai and colleagues performed transcriptomic and chromatin immunoprecipitation analysis, showing that PBRM1 directly bound to the Vimentin promoter. This evidence is key to understand how an intact SWI/SNF complex is required for the maintenance of an epithelial lineage. Further studies are required to understand how *Pbmr1*, and, broadly, the SWI/SNF, regulates cell-state–specific transition in PDAC, such as the increased expression of squamous markers, as observed by Kawai et al. (7) It is noteworthy that these results are in line with recent evidence suggesting that a proficient SWI/SNF complex is required for cell identity specification in postmitotic cells through chromatin bookmarking (10) and that dysregulation of the complex leads to lineage infidelity and phenotypic entropy. It is still to be determined if the function of SWI/SNF components in the context of cell-state transitions and tumor progression are redundant or whether some degree of subunit specificity exists. For example, recent clinicogenomic data of large patient cohorts suggest that alterations in *SMARCB1*, another SWI/SNF member, are mutually exclusive with *KRAS* truncal mutations (2). Moreover, the mechanisms by which *Pbrm1* safeguards ductal and epithelial lineages in PDAC are yet to be fully elucidated. In the future, molecular and functional studies need to address these questions and provide a mechanistic explanation of the role of the SWI/SNF complex in the emergence of cancer cells with high degrees of cell plasticity. These findings highlight the tumor suppressor role of *Pbrm1*, indicating its contribution to the aggressive nature of PDAC and its poor outcomes.

## Conclusions

Kawai and colleagues provided solid in vivo evidence and cross-species analysis of PDAC, suggesting that *PBRM1* has a role during tumor progression in promoting lineage infidelity and enabling the emergence of high-grade histologies. This study confirms a role for EMT in the emergence of aggressive disease and potentially reveals specific vulnerabilities of *PBRM1*-deficient tumors (7). Overall, *PBRM1* is a gatekeeper of epithelial identity in PDAC and its loss unlocks the full evolutionary potential of pancreatic cancer.

## Version 1 06/02/2025

Electronic publication
